# Colocalization of endogenous TNF with a functional intracellular splice form of human TNF receptor type 2

**DOI:** 10.1186/1476-9255-2-7

**Published:** 2005-07-04

**Authors:** Christoph Scherübl, Wulf Schneider-Brachert, Stephan Schütze, Thomas Hehlgans, Daniela N Männel

**Affiliations:** 1 University of Regensburg, Institute of Immunology; 2 Institute of Medical Microbiology and Hygiene, D-93042 Regensburg; 3 University Hospital of Schleswig-Holstein Campus Kiel, Institute of Immunology, D-24105 Kiel, Germany

## Abstract

**Background:**

Tumor necrosis factor (TNF) is a pleiotropic cytokine involved in a broad spectrum of inflammatory and immune responses including proliferation, differentiation, and cell death. The biological effects of TNF are mediated via two cell surface TNF receptors: p55TNFR (TNFR1; CD120a) and p75TNFR (TNFR2; CD120b). Soluble forms of these two receptors consisting of the extracellular domains are proteolytically cleaved from the membrane and act as inhibitors. A novel p75TNFR isoform generated by the use of an additional transcriptional start site has been described and was termed hicp75TNFR. We focused on the characterization of this new isoform as this protein may be involved in chronic inflammatory processes.

**Methods:**

Cell lines were retroviraly transduced with hp75TNFR isoforms. Subcellular localization and colocalization studies with TNF were performed using fluorescence microscopy including exhaustive photon reassignment software, flow cytometry, and receptosome isolation by magnetic means. Biochemical properties of the hicp75TNFR were determined by affinity chromatography, ELISA, and western blot techniques.

**Results:**

We describe the localization and activation of a differentially spliced and mainly intracellularly expressed isoform of human p75TNFR, termed hicp75TNFR. Expression studies with hicp75TNFR cDNA in different cell types showed the resulting protein mostly retained in the trans-Golgi network and in endosomes and colocalizes with endogenous TNF. Surface expressed hicp75TNFR behaves like hp75TNFR demonstrating susceptibility for TACE-induced shedding and NFκB activation after TNF binding.

**Conclusion:**

Our data demonstrate that intracellular hicp75TNFR is not accessible for exogenously provided TNF but colocalizes with endogenously produced TNF. These findings suggest a possible intracellular activation mechanism of hicp75TNFR by endogenous TNF. Subsequent NFκB activation might induce anti-apoptotic mechanisms to protect TNF-producing cells from cytotoxic effects of TNF. In addition, the intracellular and not TACE-accessible splice form of the hp75TNFR could serve as a pool of preformed, functional hp75TNFR.

## Background

TNF is a pleiotropic cytokine involved in a broad spectrum of inflammatory and immune responses including proliferation and cytotoxicity in a variety of different cell types [1]. Two distinct receptor molecules with an apparent molecular mass of 55 kDa (p55TNFR, TNFR type 1) and 75 kDa (p75TNFR, TNFR type 2) have been identified and their corresponding cDNAs cloned [2-5]. The p55TNFR is expressed rather constitutively on a broad spectrum of different cell types and has been shown to mediate most of the commonly known biological effects of TNF [6,7]. In contrast, expression of the p75TNFR seems to be modulated by various stimuli. However, there are only a few cellular responses that can be attributed exclusively to signalling via the p75TNFR, e.g. proliferation of NK cells [8], B cells [9], thymocytes [10], and mature T cells [11], and GM-CSF secretion of T lymphocytes [12]. Moreover, the p75TNFR has been shown to be preferentially activated by membrane-bound TNF [13]. Although the intracellular domains of the two TNFR show only little similarity they share activities like NFκB activation. While p55TNFR is capable of mediating these effects when expressed at physiologically relevant levels, induction of NFκB via the p75TNFR alone was observed only in cells overexpressing this receptor subtype [14,15].

The extracellular domain of both TNFR is suceptible to proteolytic cleavage. Agents like the natural ligand TNF, LPS, anti CD3- antibodies, and other stimuli induce a rapid receptor shedding in several cell types including macrophages, T- cells, and granulocytes [16-19].

High levels of soluble p75TNFR are found in sera of patients suffering from cancer [20], HIV [21], sepsis [22], and several autoimmune diseases like rheumatoid arthritis [23] and systemic lupus erythematodes [24]. Expression of a secreted soluble p75TNFR isoform, generated by differential splicing, was recently described to be elevated in rheumatoid arthritis [25].

A novel p75TNFR isoform generated by the use of an additional transcriptional start site has been described and was termed hicp75TNFR [26]. Exon 1 that contributes to the signal peptide in human p75TNFR is replaced by Exon1a consisting of an Alu element which was exonized during evolution in both mouse and human[27]. Several cell lines e.g. activated macrophages express hicp75TNFR in parallel to hp75TNFR [26] and hicp75TNFR mRNA upregulation was observed in mouse livers after injection of LPS in mice sensitized with D-GalN (unpublished observation). While the relevance of soluble TNFR as inhibitory molecules is generally accepted the function of an intracellular TNFR in inflammatory processes remains elusive. In this study we determined the localization of hicp75TNFR and tested possible ways of activation by exogenous and endogenous TNF.

## Methods

### Cell culture and reagents

HEK 293 cells and NIH 3T3 cells were maintained in Dulbeccos's Mod Eagle Medium (Invitrogen, Karlsruhe, Germany) supplemented with 10% heat- inactivated fetal calf serum (PAN Biotech GmbH, Aidenbach, Germany) and 50 μg/ml gentamycin (PAA Laboratories, Linz, Austria). p55TNFR and p75TNFR double-deficient fibroblasts (TNFR1/2) were generated in our lab by simian virus 40 large T-immortalization of murine fibroblasts from TNFR1 and TNFR2 double knock-out mice [28]. L929 cells and TNFR1/2 knock-out fibroblasts were grown in RPMI 1640 medium (Sigma-Aldrich Chemie GmbH, Deisenhofen, Germany) supplemented with 10% heat- inactivated fetal calf serum and 50 μg/ml gentamycin. The human p75TNFR-specific monoclonal mouse antibody 80M2 and rabbit serum 80M [29] were kindly provided by P. Scheurich (University of Stuttgart, Germany). The mouse monoclonal anti-myc antibody (9E10) was purchased from Invitrogen (Karlsruhe, Germany). Polyclonal rabbit IgG antibodies anti-human TNF were from Santa Cruz Biotechnology, Inc. (Santa Cruz, CA, USA). Secondary antibodies for immunostaining: rabbit anti-mouse FITC was from DakoCytomation GmbH (Hamburg, Germany), and goat anti-rabbit TRITC from Sigma-Aldrich.

### Transfection and transduction

NIH 3T3 cells were transiently transfected with SuperFect Transfection Reagent (Quiagen, Hilden, Germany) according to the manufacturers instruction. In order to produce retrovirus containing supernatant HEK 293 cells were transiently cotransfected with the packaging construct pCl-10 A1 [30] and the retroviral vector pQCXIP (BD Biosciences Clontech, CA, USA) containing the gene of interest using the HEPES-buffered saline calcium phosphate method [31]. Medium was replaced 5 h post transfection. Supernatants were collected after 2 days and filtered through a low-protein binding 0.45 μm pore-size filter (Acrodisc Syringe filter, PALL Corporation, MI, USA) in the presence of 8 μg/ml polybrene (Sigma-Aldrich). Target cells were infected during 2 days by changing the virus containing medium in 12 h steps and afterwards selected for positivity either by FACS-sorting in the case of fluorescent cells or by puromycin (Sigma- Aldrich) selection.

### Fluorescence microscopy

Transduced NIH 3T3 cells were seeded in Lab- Tek II Chamber Slide systems (Nunc GmbH & Co. KG, Wiesbaden, Germany). The following day they were fixed 15 min with 4% paraformaldehyde in phosphate-buffered saline and permeabilized with 0.1% Triton X-100 for 5 min. After blocking with 1% bovine serum albumin in phosphate-buffered saline primary antibody (2 μg/ml) was added followed by a FITC or TRITC labelled secondary antibody. Before mounting the coverslide with MoBiGLOW Mounting Medium (MoBiTec GmbH, Göttingen, Germany) nuclei were stained with Dapi (Sigma-Aldrich). Staining of the different compartments and nuclei was performed on living cells by using Hoechst 33342, ER-Tracker Blue-White DPX, GolgiTracker BODIPY TR C_5_-ceramide, MitoTracker Red CMXRos, LysoTracker Red DND-99 (all from Molecular Probes, Eugene, OR, USA) according to the manufacturers instruction. The vector ENDO-eCFP was purchased from BD Biosciences Clontech (CA, USA). After washing with phosphate-buffered saline cells were fixed with 4% paraformaldehyde in phosphate-buffered saline at 4°C for 5 min followed by 10 min at room temperature. Coverslides were mounted with MobiGLOW Mounting. Fluorescent optical sections for each color were obtained with a conventional Zeiss Axiovert microscope equipped with a piezoelectric z-axis focus device (Physik Instrumente, Waldbronn, Germany). Images were taken with a charge-coupled device (CCD) camera (4096 levels of gray, -15°C peltier cooled, Princeton Instruments, Trenton, USA) and processed by MetaMorph software (Universal Imaging Corp., West Chester, USA). The light haze contributed by fluorescent-labelled structures located above and below the plane of optimal focus was mathematically reassigned to its proper place of origin (EPR, Exhaustive Photon Reassignment software Scanalytics, Massachusetts, USA) after accurate characterization of the blurring function of optical system (point spread function, PSF). During restoration, EPR used the PSF image data to refocus light and haze in the raw specimen image.

### ELISA

To detect soluble extracellular domains of either hicp75TNFR or hp75TNFR the human p75TNFR (80 kDa) Module Set (Bender MedSystems, Vienna, Austria), and human sTNFRII/TNFRSF1B Duo Set ELISA development (R&D Systems, MN, USA) were used. Cells (4 × 10^4^) were seeded in triplicates into 48-wells culture plates, and incubated either with hTNF, medium alone, or the TACE-inhibitor TAPI-0 (Biomol, Hamburg, Germany) in concentrations as indicated. After 24 h supernatants were collected and tested according to the manufacturers instructions. The OD of ABTS was determined at 405 nm.

### Flow cytometry

Expression of transduced hp75TNFR isoforms on the cell surface was detected by flow cytometry on a FACStar Plus (Becton Dickinson, San Jose, CA, USA) using the PE-coupled specific rat anti-human p75TNFR monoclonal antibody and PE-labelled rat IgG2b (both BD, Heidelberg, Germany) as isotype control. For FACS analysis cells (1 × 10^6^/tube) were blocked with PBS containing 10% heat inactivated FCS for 30 min on ice and then incubated with 20 μl of antibody solution on ice for 30 min. The YFP-tagged fusion proteins were directly detectable in FL-1 without any additional staining step. YFP-positive cells were sorted using a FACStar Plus. For intracellular staining FIX&PERM (Caltag, Hamburg, Germany) was used.

### Western Blot and Immunoprecipitation

Cells (4 × 10^6^) were seeded in 10 cm dishes and grown overnight. After 24 h cells were washed with ice-cold PBS and lysed in 1 ml of buffer (150 mM NaCl, 50 mM Tris· HCl, pH 7.4,1 mM EDTA, 1% Triton X-100, NP-40 1%, Na-deoxycholate 0,25%) containing a mixture of protease inhibitors (Complete™ EDTA-free tablets, Roche, Mannheim, Germany). After centrifugation lysates were precleared for 4 h in 20 μl of protein G-Sepharose (Amersham Biosciences, Uppsala, Sweden), and immunoprecipitated with 80M2 (10 μg/ml) a mouse monoclonal antibody against the extracellular domain of human p75TNFR [29] and 20 μl of protein G-Sepharose for 16 h at 4 C. Pellets were washed three times in PBS, resolved on 8% SDS-PAGE under reducing conditions, and transferred to PVDF membranes. These membranes were blocked in 1% non-fat milk powder and detected with a rabbit polyclonal antibody (0.1 μg/ml) H-202 (Santa-Cruz Biotechnology, Inc., CA, USA) binding to the intracellular domain of hp75TNFR isoforms, to specifically detect the full-length protein. As secondary antibody a goat anti-rabbit IgG-HRP (Dilution 1:2000, Sigma- Aldrich) was used. The blots were then incubated with HRP substrate (enhanced chemiluminescence substrate NOWA solution A and B; MoBiTec GmbH, Germany) and developed by exposure to film (Hyperfilm; Amersham Biosciences).

### Cloning

PCR amplification was performed using sense primers for hicp75TNFR-myc (5'-AAAGGATCCCCCATGGCGAAACCCCTC-3') and for hp75TNFR-myc (5'-AAAGGATCCCCCATGGCGCCCGTCGCCGTC-3') and antisense primers for both (5'GGGCTCGAGTCACAGATCCTCTTCTGAGAT-3'). The template in each case was the myc-tagged cDNA in pcDNA 3.1 hygro (Invitrogen, Karlsruhe, Germany). The resulting PCR products were cloned using BamHI/xhoI into a modified proviral vector, pQCXIP (BD Biosciences Clontech, CA, USA) containing an additional xhoI site on the 3' end of the multiple cloning site. To create eYFP-tagged fusion proteins the whole MCS of pQCXIP (BD Biosciences Clontech, CA, USA) was substituted by the MCS and eYFP coding fragment from pEYFP- N1 (BD Biosciences Clontech, CA, USA). In this modified vector both myc-tagged cDNA's were cloned using EcoRI/BamHI in frame with eYFP using sense primers for hicp75TNFR-myc (5'-CCGAATTCCCAGCCATGGCGAAACCCCTC-3') and for hp75TNFR-myc (5'-CCCAAGCTTGAATTCCCAGCCATGGCGCCCGTCGCCGTC-3') and antisense primers for both (5'CGGGATCCCGCAGATCCTCTTCTGAGATG-3'). All expression constructs have been verified by sequencing.

### Isolation of magnetically labelled TNF receptosomes

To isolate TNF plasma membrane receptors after ligand binding and internalization biotinylated TNF (Fluorokine-Kit, R&D Systems, Wiesbaden, Germany) and magnetically labelled 50 nm MACS Streptavidin Microbeads (Miltenyi Biotec, Bergisch Gladbach, Germany) were used and the receptosomes isolated by magnetic means after different time points as described recently [32]. Cells were incubated in a total volume of 250 μl cold Dulbecco's Modified Eagles Medium (DMEM) supplemented with 25 Mm HEPES (Invitrogen, Karlsruhe, Germany) with 100 μl (400 ng) of biotinylated TNF for 1 h on ice. Thereafter, 200 μl of the MACS Streptavidin Microbeads solution was added and cells were incubated for 1 h on ice. TNF receptor clustering and formation of magnetized TNF receptosomes was achieved by incubation at 37°C for different times. The labeled cells were mechanically homogenized using steel beads in a 0.25M sucrose buffer, supplemented with 0.015M HEPES, 100 mg/L MgCl_2_, pH 7.4 and the Protease Inhibitors Set from Roche Diagnostics (Mannheim, Germany) at 4°C. A postnuclear supernatant was submitted to magnetic separation of TNF receptosomes in a high-gradient magnetic field generated in a custom-built free-flow magnetic chamber (German patent held by S. Schütze).

### Characterization of TNF receptosome-associated proteins

Receptosome proteins were separated by SDS-PAGE and analyzed by Western blotting for signature proteins of endocytosis and vesicular trafficking by antibodies against clathrin, (Transduction Lab., Lexington, UK), Rab5, Vti1b (StressGene Biotec. Corp. Victoria, Canada), and cathepsin D (Calbiochem-Novabiochem GmbH, Germany) as described [32].

### Affinity chromatography

Purified rhTNF (BASF, Ludwigshafen, Germany) was coupled to CNBr-activated Sepharose 4B according to the protocol provided by Amersham Biosciences (capacity 16–32 mg/g) with the following modifications: The gel was swollen with 1 mM of HCl and washed three times for 5 min each, using a total of 200 ml of 1 mM HCl/g of gel. rhTNF was dissolved at 7.5 mg/3 ml of phosphate-buffered saline and dialyzed three times against 500 ml of 0.1M NaHCO%_3_, pH 8.3, 0.5M NaCl. The volume of the ligand was made up to 5 ml with the same buffer and mixed with the washed gel (2.5 mg of rhTNF/ml of swollen gel slurry) by rotating gently overnight at 4°C. Excess ligand was washed away with the same buffer, and the remaining active groups were blocked with 0.1M Tris-HCl, pH 8.0 for 2 h at room temperature. The gel was then washed with 3 cycles of alternating pH, using 0.1M sodium acetate buffer, pH 4.0, and 0.1M Tris-HCl, pH 8.0, each containing 0.5M NaCl. The conjugate was stored in PBS at 4°C. Clarified supernatant from transduced TNFR1/2 knock-out fibroblasts was precipitated with ammonium sulfate. The pellet was resuspendet in PBS, dialysed against PBS and loaded onto a rhTNF affinity column by using FPLC (Bio-Rad Laboratories, Inc., CA, USA). After washing with PBS, the bound material was eluted with 100 mM glycine, 100 mM NaCl, pH 2.6, into microcentrifuge tubes containing 1M Tris-HCl, pH 7.5. Fractions were analyzed by 8% SDS-PAGE and following Coomassie Blue staining or Western blotting.

### Determination of cytotoxic activity of TNF on L929 cells

Cells (L929) were seeded in 96-well microtiter plates at 2.5 × 10^4 ^cells/well. After 24 h serial dilutions of rhTNF were added in the presence of actinomycin D (2 μg/ml). After 24 h, cell viability was assessed by adding MTT (Sigma- Aldrich) for 4 h. Cells were lysed with SDS and OD were determined at 450 nm.

## Results

### Localization of tagged human p75TNFR isoforms

**Figure 1:**
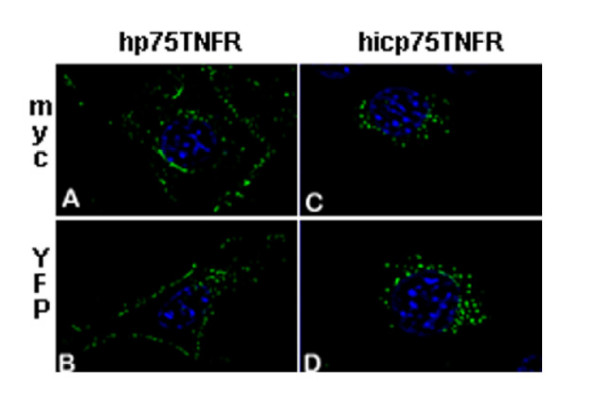
**Localization of tagged hp75TNFR and hicp75TNFR molecules in transduced NIH 3T3 cells. **Cells transduced either with myc-tagged (A;C) or YFP-tagged (B;D) hp75TNFR (A;B) or hicp75TNFR (C;D) were analyzed microscopically. For detection of YFP the cells were fixed and analyzed. For detection of the myc-tag cells were fixed and permeabilized followed by incubation with a monoclonal mouse anti-human c-myc (9E10) and a secondary anti-mouse FITC antibody. While hp75TNFR staining is mainly found on the plasma membrane, hicp75TNFR staining is perinuclear, punctuated, and distributed throughout the cytoplasm. Nuclei: Dapi (A;C), Hoechst 33342 (B;D).

To obtain a detailed picture of the subcellular localization of the hicp75TNFR we used YFP-tagged receptor constructs and compared the staining with compartment-specific fluorescent trackers. As shown in Fig. 1 the big barrel structure of the YFP protein is not altering the staining pattern of the hp75TNFR protein when compared to the relatively small myc-tag. While the hp75TNFR stained in the manner of a typical membrane-localized protein, the hicp75TNFR exhibited a punctuated, vesicle-like pattern localized perinuclearly and throughout the cytoplasm. In addition, we also transfected cells with the corresponding cDNAs both transiently and stably to rule out potential epiphenomena created by viral transduction. The data obtained by transfection were in line with those obtained by transduction (data not shown).

### Subcellular localization of human icp75TNFR

**Figure 2:**
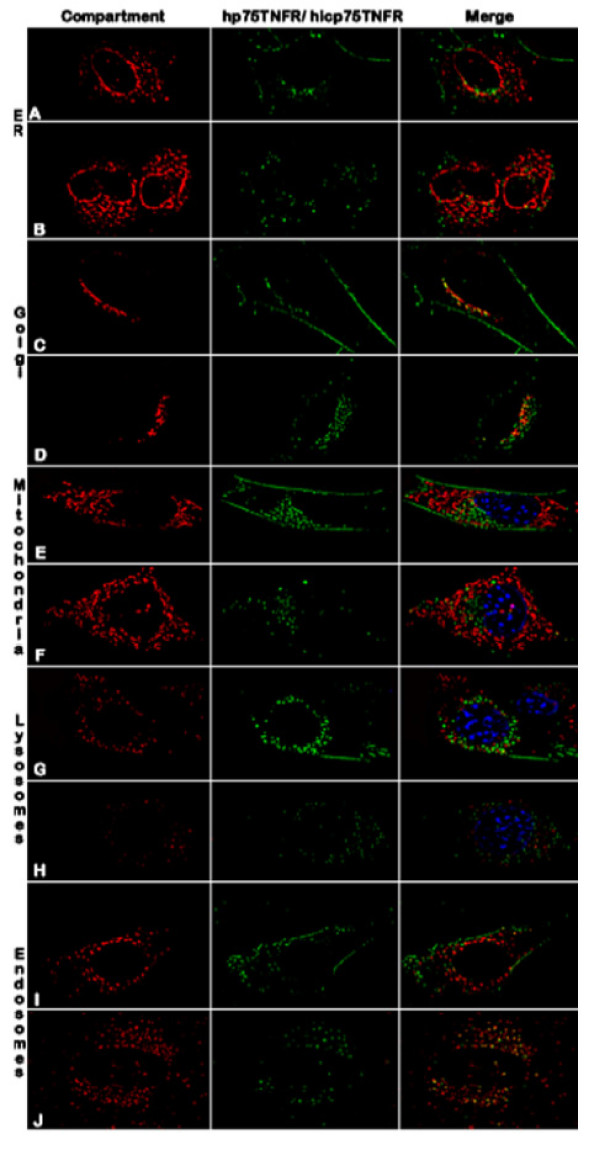
**Localization of hp75TNFR isoforms in transduced NIH 3T3 cells. **Cells transduced either with YFP-tagged hp75TNFR (A; C; E; G; I) or YFP-tagged hicp75TNFR (B; D; F; H; J) were costained with ER-Tracker (A; B), Golgi-Tracker (C; D), Mito-Tracker (E; F), Lyso-Tracker (G; H) and ENDO-eCFP (I; J). While hp75TNFR staining is mainly found on the plasma membrane and in the Golgi apparatus, hicp75TNFR shows no plasma membrane staining and colocalizes with the Golgi apparatus and endosomal compartments of the trans-Golgi network.

When TNFR localization was compared to different cellular compartments none of the two hp75TNFR variants co-localized with the ER (Fig. 2A; 2B), mitochondria (Fig. 2E; 2F), or lysosomes (Fig. 2G; 2H). Both receptor forms colocalized with the Golgi apparatus (Fig. 2C; 2D). A clear discrimination of colocalization with perinuclear budded endosomes resembling the trans-golgi network (TGN) was observed when hp75TNFR staining was compared to hicp75TNFR staining. Whereas the hp75TNFR was quickly guided through this compartment (Fig. 2I) it seemed that most of the hicp75TNFR-YFP molecules were stored in endosomal structures as documented by the strong colocalization of hicp75TNFR with ENDO-eCFP (Fig. 2J).

### Cell surface expression of human p75TNFR isoforms

Although transfected hicp75TNFR was not detectable microscopically, we used flow cytometry to determine whether hicp75TNFR becomes detectable on the cell membrane after transduction.

**Figure 3:**
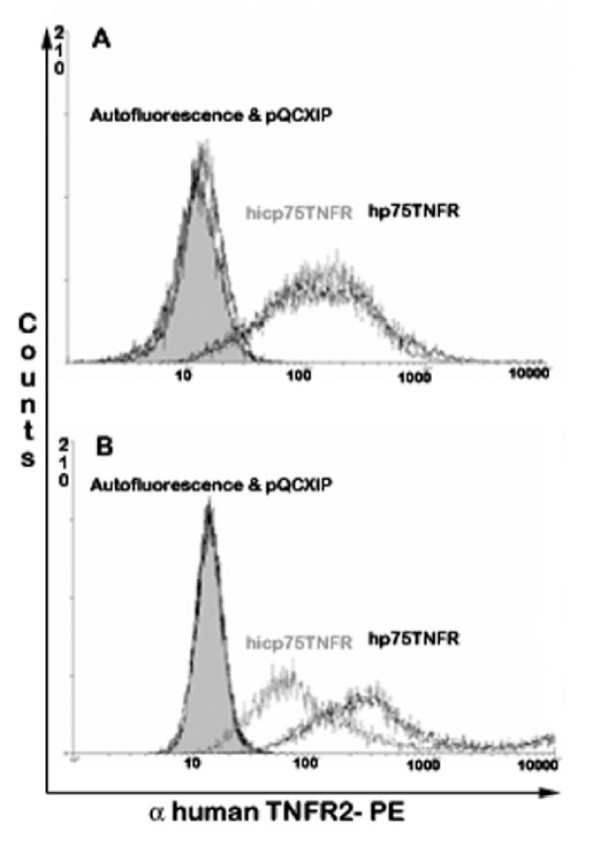
**Cell surface expression of hp75TNFR isoforms on transduced L929 cells. **Expression of hp75TNFR isoforms on L929 cells either transduced with control vector (shaded peak), hp75TNFR (black line) or hicp75TNFR (gray line) was analyzed by flow cytometry with (A) or without (B) permeabilization. After permeabilization the same number of epitopes were accessible in both cell lines. Without permeabilization cells expressing hp75TNFR present more epitopes on the cell surface.

Fig. 3A shows that both L929 cell lines either transduced with hp75TNFR or hicp75TNFR expressed the same number of the respective hp75TNFR isoform. After fixation and permeabilization a specific PE-labelled antibody against an epitope present on both hp75TNFR and hicp75TNFR stained the transduced L929 cell lines with equal intensities (Fig. 3A) demonstrating that the used antibody has the same affinity to both isoforms and that the cells express a comparable amount of this epitope. Without cell permeabilization hp75TNFR isoforms exposed on the outer cell membrane became detectable which is in line with the microscopic observations (Fig. 2). Surprisingly, hicp75TNFR molecules were also stained on the cell membrane, however to a lower extent than hp75TNFR molecules (Fig. 3B).

### Soluble ectodomain of human p75TNFR isoforms

Soluble forms of both TNF receptors are described, consisting of the shed extracellular domains of the p55TNFR or p75TNFR, respectively. The release can be enhanced by stimulation with TNF, LPS, or phorbol ester and can be inhibited by TAPI (TNF-α processing inhibitor). This event mainly takes place on the cell surface and is caused by metalloproteases [33].

**Figure 4:**
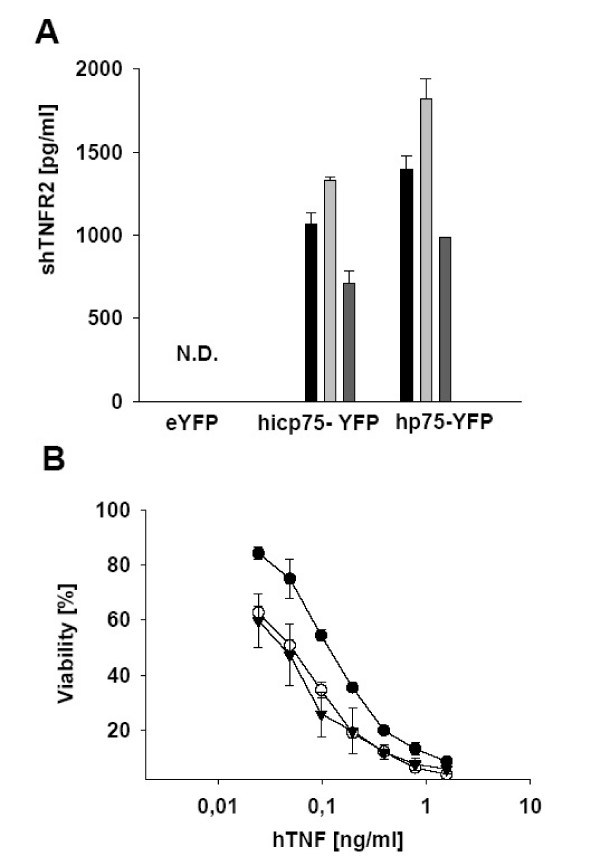
**Shedded ectodomain of hp75TNFR isoforms. **(A)The extracellular domain of both hp75TNFR isoforms is released constitutively (black bars) into the supernatant of L929 cell either transduced with hp75TNFR or hicp75TNFR, respectively. Shedding is increased by rhTNF (6 ng/ml; light gray bars) and attenuated by TAPI (100 nM, dark gray bars). (B)The extracellular domain of hicp75TNFR is bioactive as shown by neutralization of rhTNF in a TNF cytotoxicity assay on L929 cells. Supernatant from L929 cells either transduced with icp75TNFR-YFP (●), or YFP alone (○) were tested. DMEM (▼) served as control medium. All values are given as mean ± S.D. of triplicate cultures. Four independent experiments gave similar results.

We tested transduced L929 cells sorted for comperable hp75TNFR-YFP and hicp75TNFR-YFP expression concerning the release of soluble receptor by ELISA (Fig. 4A). L929 cells transduced with hp75TNFR-YFP as well as hicp75TNFR-YFP-transduced L929 cells released soluble hp75TNFR into the supernatant (Fig. 4A). More soluble hp75TNFR was measured than soluble hicp75TNFR. In the supernatant from control L929 cells transduced with YFP no soluble TNFR was found. This data indicate that hicp75TNFR is less affected by shedding. In both cell lines shedding was TACE-dependent as indicated by its inducibility with rhTNF and its attenuation with the specific TACE inhibitor TAPI. TNF was increasing the shedding by 25% in the hicp75TNFR tranductants and by 30% in the hp75TNFR transductants. TAPI reduced rhTNF induced shedding to 58% in the hicp75TNFR transductants and to 60% in the hp75TNFR transductants, showing that activitiy of TACE was equal in both cell lines and therefore not the reason for the lower soluble hicp75TNFR in the supernatant.

When the soluble hp75TNFR from the supernatant of hicp75TNFR-YFP transduced L929 cells was tested in a TNF cytotoxicity assay on L929 cells (Fig. 4B) the inhibitory activity of the shedded extracellular domain of the hicp75TNFR was demonstrated. This indicated that the ectodomain of hicp75TNFR is biologically active in binding and neutralizing TNF.

### Biochemical characterization of human icp75TNFR

The Exon 1a of hicp75TNFR does not encode a typical signal peptide and is, therefore, not cleaved off in the biosynthetic process by signal peptidases in contrast to the sequence encoded by Exon 1 of hp75TNFR. To test whether this results in a different apparent molecular weight, we isolated full length protein from total lysates of NIH 3T3 cells either transduced with hp75TNFR or hicp75TNFR, respectively, by immunoprecipitation with a monoclonal mouse antibody raised against the extracellular domain of hp75TNFR (80M2).

**Figure 5:**
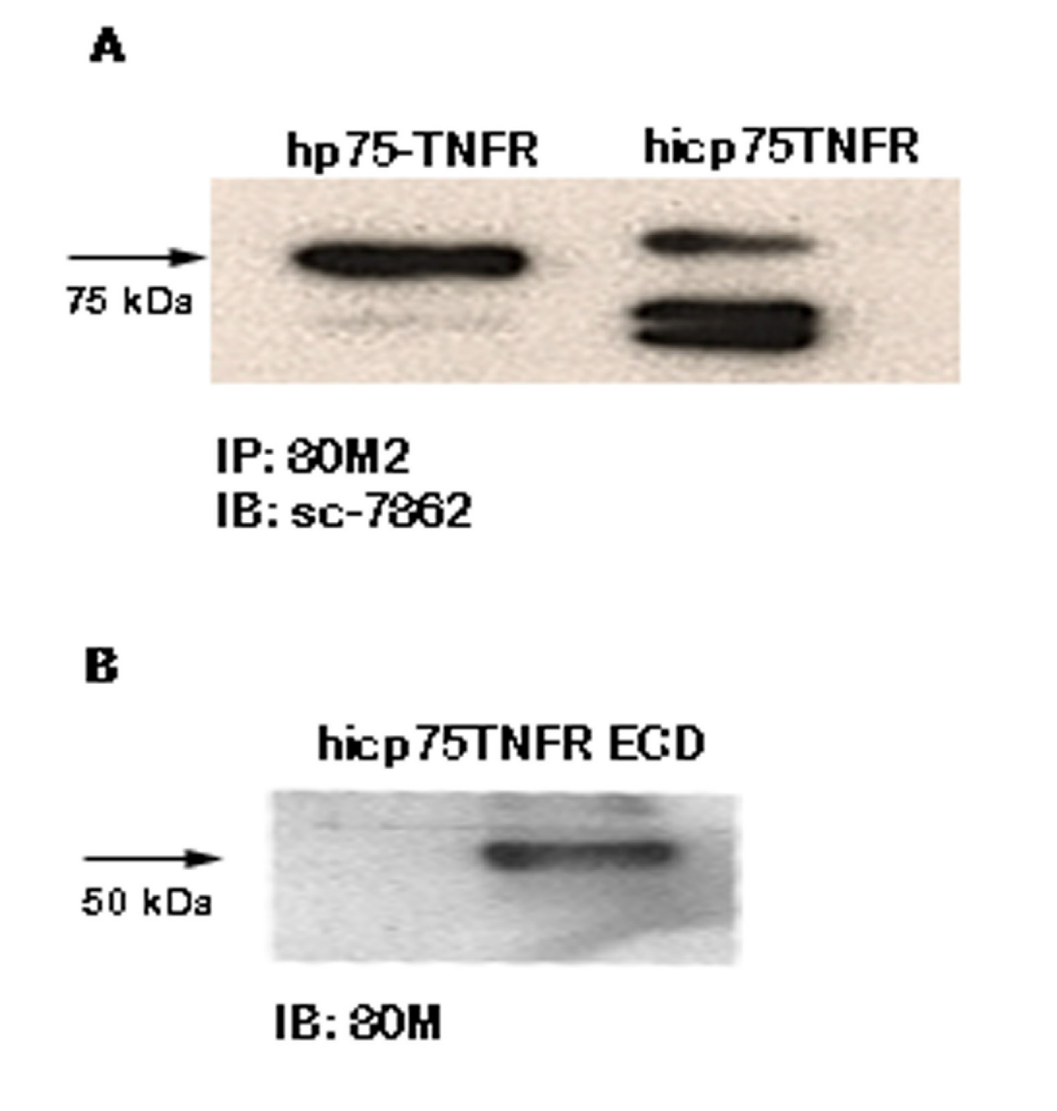
**Biochemical characterization of hicp75TNFR.**(A) The hp75TNFR from NIH3T3 cells either transduced with hp75TNFR or hicp75TNFR, respectively, were immunoprecipitated with monoclonal antibodies against the extracellular domain of hp75TNFR (80M2) and stained after blotting with antibodies to the intracellular domain on hp75TNFR (sc-7862). (B) Affinity purified soluble hicp75TNFR from supernatant of transduced TNFR1/2 knock-out fibroblasts was stained after blotting with a rabbit serum against the extracellular domain of hp75TNFR (80M).

Immunodetection was done with a polyclonal rabbit IgG fraction (sc-7862) detecting the intracellular domain of hp75TNFR. The full-length mature protein resulting from hicp75TNFR cDNA has 85 kDa, which is about 10 kDa more than the apparent molecular mass of hp75TNFR (Fig. 5A). In addition, the hp75TNFR also appears as a faint band with about 50 kDa as already described earlier as a possibly differently glycosylated hp75TNFR species [34,35]. A prominent double band was also stained in the case of hicp75TNFR at about 50 kDa possibly indicating different molecular masses for icp75TNFR molecules resulting from potential O-glycosylation sites at threonin 7 and serin 11 of the hicp75TNFR-specific exon 1a (unpublished observation).

To determine whether the difference in the apparent molecular weight is due to the presence of the additional presequence in the extracellular domain, the soluble extracellular domain of hicp75TNFR was purified from cell culture supernatant by affinity chromatography. To avoid contamination with shed mouse TNFR we used TNFR1/2 knock-out fibroblasts transduced with hicp75TNFR. The purified soluble hicp75TNFR was stained with anti-hp75TNFR antiserum (80M) and showed a single band at about 50 kDa (Fig. 5B). Since the soluble hp75TNFR has been published with an apparent molecular weight of 40kDa [36,37] these data indicate that the higher molecular mass of hicp75TNFR might result from the additional 24 amino acids at the N-terminal end encoded by Exon1a and/or different glycosylation.

### Activation of human icp75TNFR

**Figure 6:**
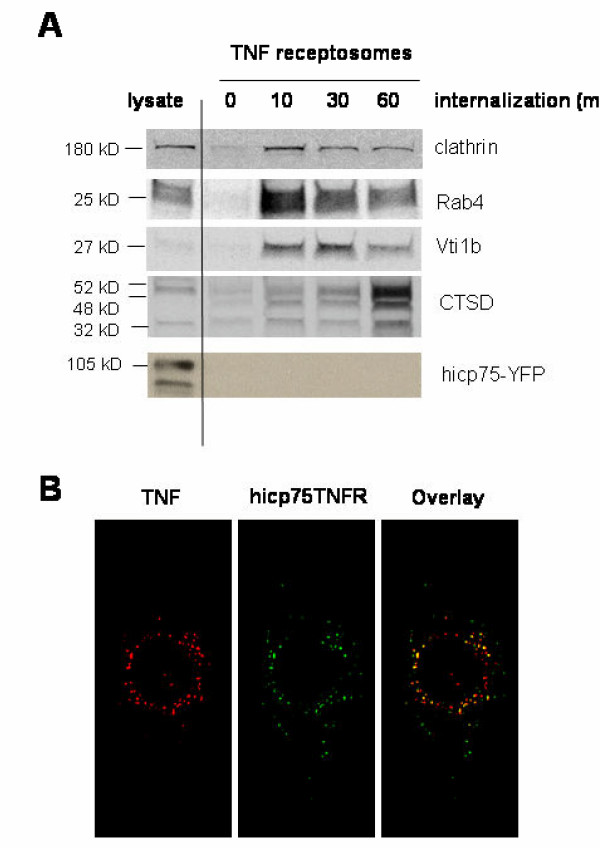
**Colocalization of hicp75TNFR with endogenous or exogenous TNF. **(A) L929 cells were transduced with hicp75TNFR-YFP and exposed to biotinylated TNF. Receptosomes were isolated after different times, subjected to SDS-PAGE and analyzed by immunostaining of blotted proteins. (B) L929 cells expressing hTNF were transduced with hicp75TNFR and stained with rabbit anti-human TNF antibodies and mouse monoclonal antibody anti-human p75TNFR (80M2).

Internalization of p55TNFR after binding of exogenous TNF has been published [38]. To test whether exogenous TNF interacts with hicp75TNFR a new method recently described by Schneider-Brachert et al. was used [32]. We isolated receptosomes at different time points in a magnetic field and followed maturation of the resulting endosomes by testing for different marker proteins recruited to the complex (Fig. 6A).

Over time after binding, clathrin as a marker for endocytosis is decreasing as well as the early endosomal marker Rab4. After 30 minutes the late endosomes fuse with vesicles from the TGN, as documented by an increasing amount of Vti1. Even though this could be the point in time when exogenous TNF could be passed from the internalized TNF-p55TNFR complex to the hicp75TNFR, no hicp75TNFR was isolated in receptosomes after 30 minutes indicating that no intracellular ligand passing is occurring. After 60 min the receptosomes end up in lysosomes indicated by Cathepsin D staining.

To evaluate a possible intracellular activation of hicp75TNFR with endogenously produced TNF L929 stably transfected with full-length, transmembrane TNF were transduced with hicp75TNFR (Fig. 6B). After double staining colocalization of human endogenous TNF and hicp75TNFR was observed in intracellular perinuclear compartments indicating that endogenous TNF could possibly activate the hicp75TNFR.

## Discussion

TNF exerts pleiotropic biological activities affecting proliferation, differentiation, or functions in a wide variety of cell types by interacting with its two distinct receptors p55TNFR and p75TNFR [17,18]. Soluble TNF receptors, generated either by proteolytic cleavage [16-19] or differential splicing [25] contribute to the balance of TNF-mediated effects by neutralization of the ligand. The biological function of a mainly intracellularly expressed isoform of hp75TNFR was not clear. Since hicp75TNFR is lacking a typical leader sequence at the N-terminus we used YFP-tagged receptor constructs to investigate whether the protein is directed to a specific intracellular compartment. Localization of the YFP-tagged hicp75TNFR was not different from the corresponding myc-tagged hicp75TNFR. In this way it was not necessary to perform permeabilization and incubation steps, that could probably alter the 3D structure of the cells in colocalization studies with compartment-specific dyes. In case of the hp75TNFR-YFP the expected pattern of a typical plasma membrane-bound receptor was seen, comparable to native p75TNFR expression in human umbilical vein endothelial cells [39].

The hicp75TNFR-YFP protein was not retained in the ER. Also, this receptor is not a mitochondrial protein as has been described by crossreaction with an anti-hp75TNFR antibody [40]. Exogenously added TNF internalizes after binding to the p55TNFR and is transported to the lysosomes [38,41]. The hicp75TNFR did not colocalize with lysosomes and was not found to interact with endocytosed exogenous TNF.

Besides being enriched in the final compartment, overexpressed cellular proteins are usually observed in the Golgi apparatus. The hicp75TNFR strongly colocalized with the Golgi apparatus and with budding endosomal vesicles of the trans-Golgi network (TGN) as indicated by the coexpression with a labelled endosomal marker. No hicp75TNFR staining was observed on the plasma membrane or any other intracellular compartment.

The limitations of sensitivity of fluorescent microscopy become obvious considering the intracellular but not membrane staining of p55TNFR by confocal microscopy. This TNFR is also predominantly seen in the TGN [39,42], even though it is a well characterized plasma membrane protein. Flow cytometrical analysis confirmed indeed that both isoforms of the hp75TNFR can be found on the cell surface with stronger expression of the hp75TNFR compared to the hicp75TNFR isoform. The hicp75TNFR expression reminds of the human transferrin receptor which is also synthesized without a typical leader sequence and, therefore, localized predominantly within the cell. Only a small amount is localized in the plasma membrane [43] where the protein acts as a receptor. It has been shown that a transmembrane domain is sufficient to translocate proteins into the membrane of the ER from where they travel to the plasma membrane via the secretory pathway [43,44].

As a direct consequence of the cell surface expression the hicp75TNFR becomes accessible to the TNF-α convertase (TACE/ADAM-17), a transmembrane disintegrin metalloproteinase of the ADAM family of proteases. The ADAM-17-dependency on shedding of the extracellular domain of hicp75TNFR is demonstrated by the TNF inducibility and reduced shedding in the presence of the specific TACE inhibitor TAPI. In general, lower amounts of soluble hicp75TNFR were detected in supernatants of transduced cells compared to soluble hp75TNFR. These data indicate that the hicp75TNFR molecules emerging on the cell membrane do not behave differently than the hp75TNFR molecules. The intracellular pool of hicp75TNFR does not seem to be affected by shedding that takes place on the cell membrane. Since after stimulation cells are depleted of TNFR [33] restoration of the cell surface with preformed functional TNFR is a very quick mechanism without the need of protein neo-biosynthesis. Such a theory of stored TNFR has also been discussed for intracellular p55TNFR molecules [45].

The soluble hicp75TNFR is biologically active and able to competitively bind TNF. Full-length p55TNFR is released in exosome-like vesicles as published recently [46]. We could exclude this possibility for the hicp75TNFR because of the clear TACE-dependent shedding and the apparent molecular weight of full length and affinity purified soluble hicp75TNFR. The soluble hicp75TNFR showed a clear band at ~50 kDa while full length hicp75TNFR appears at ~85 kDa, which is in both cases about 10 kDa more than the respective form of the hp75TNFR [37]. This increased molecular weight can be explained by the additional N-terminal sequence of 24 amino acids encoded by Exon 1a.

Addressing the question whether exogenous TNF could possibly activate the hicp75TNFR we used the elegant method of receptosome isolation [32]. The results show that internalized TNF was not passed over from the internalized p55TNFR to hicp75TNFR. Due to higher affinity of soluble TNF to the p55TNFR than to the p75TNFR [47] such passing over was rather unlikely. On the other hand, the affinity of hp75TNFR to membrane-bound TNF is relatively high [10]. Therefore, the second possibility of interaction of endogenous TNF with hicp75TNFR was tested using L929 cells stably expressing 26 kDa pre-TNF and transduced with hicp75TNFR. Overexpression of both ligand and receptor became necessary because TNF-producing cells lines such as macrophages could not be transduced efficiently with hicp75TNFR expression constructs. The distribution pattern of overexpressed endogenous TNF is similar to the TNF produced by macrophages upon LPS stimulation [48]. Clear colocalization of endogenous TNF and intracellularly localized hicp75TNFR was detected. Activation of hicp75TNFR by endogenous TNF in the TGN is difficult to unequivocally demonstrate due to the presence of soluble TNF and cell membrane-localized hicp75TNFR in this system. For intracellular p55TNFR it has already been shown that activation did not occur by exogenously added TNF [42]. Parallel expression of ligand and receptor in the same cell could lead to intracellular complexes that are rapidly degraded [49]. Alternatively, intracellular activation of the hicp75TNFR by endogenous TNF cannot be excluded.

Expression of TNF as well as hicp75TNFR by cells such as activated macrophages could lead to the activation of intracellular NFκB pathways and to the expression of NFκB-dependent anti-apoptotic proteins. The importance of NFκB in preventing apoptosis has clearly been demonstrated as fibroblasts of p65/RelA-deficient mice are highly susceptible for TNF induced apoptosis [50] and cells expressing a dominant negative mutant of IκB are also very sensitive to TNF-induced apoptosis [51,52]. Therefore, such an intracellular activation mechanism of hicp75TNFR by endogenous TNF with subsequent NFκB-dependent activation of anti-apoptotic mechanisms might protect TNF-producing cells from cytotoxic effects of TNF. As a result, survival of activated immune cells could thus become sustained.

Intracellular activation of the p75TNFR by endogenous TNF seems particularly interesting in the light of the recent publication by Kim and The [53] demonstrating costimulatory function of the p75TNFR activation for T cell activation. Thus, TNF not only serves as a central mediator of innate immunity by activating the p55TNFR but also by stimulation of the p75TNFR contributes to the induction of adaptive immune responses.

In conclusion, the results of this study show that the alternative splice variant of human p75TNFR is strongly retained in the TGN where it could function as a storage pool of preformed p75TNFR that is not affected by shedding. Upon emerging on the cell surface hicp75TNFR is functionally not longer distinguishable from hp75TNFR. The consequences of hicp75TNFR colocalization with endogenous TNF for hicp75TNFR signalling in TNF producing cells remains to be analyzed.

## Conclusion

The results of this study show that the alternative splice variant of the human p75TNFR is strongly retained in the TGN where it colocalizes with endogenous TNF. The consequences of this colocalization for hicp75TNFR signalling in TNF producing cells remains to be analyzed. Upon emerging on the cell surface hicp75TNFR is functionally not longer distinguishable from hp75TNFR. The hicp75TNFR could function as a storage pool of preformed p75TNFR that is not affected by shedding. Potential intracellular activation of hicp75TNFR by endogenous TNF could lead to elevated NFκB levels and eventually protect TNF-producing cells from TNF-induced cell death.

## Competing interests

The author(s) declare that they have no competing interests.

## Authors' contributions

CS participated in experimental design, carried out most experimental procedures and put together the manuscript. WS-B established the protocol for retroviral transduction and provided essential vectors. SS carried out the receptosome isoloation and the corresponding western blots. TH participated in experimental design, cloning and discussions. DNM participated in study design and coordination and helped to draft the manuscript. All authors read and approved the final manuscript.
